# Physical home-learning environments of traditional and non-traditional students during the COVID pandemic: exploring the impact of learning space on students’ motivation, stress and well-being

**DOI:** 10.1186/s40561-023-00222-4

**Published:** 2023-01-20

**Authors:** Sonja Brachtl, Christina Ipser, Filiz Keser Aschenberger, Sabrina Oppl, Stefan Oppl, Emre Kevin Pakoy, Gregor Radinger

**Affiliations:** 1 Department for Continuing Education Research and Educational Technologies, University for Continuing Education Krems, Dr.-Karl-Dorrek-Straße 30, 3500 Krems, Austria; 2 Department for Building and Environment, University for Continuing Education Krems, Dr.-Karl-Dorrek-Straße 30, 3500 Krems, Austria; 3 Teaching Innovation and Digital Competence Development, University for Continuing Education Krems, Dr.-Karl-Dorrek-Straße 30, 3500 Krems, Austria; 4 Institute of Business Informatics - Communications Engineering, Johannes Kepler University Linz, Altenbergerstraße 69, 4040 Linz, Austria

**Keywords:** Home learning environment, Distance learning, COVID-19 pandemic, Physical learning space, Well-being, Traditional students, Non-traditional students

## Abstract

When the COVID-19 pandemic forced higher education institutions to implement their programs in an online setting, different groups of students were influenced to different extents. In many cases, the main locus of learning moved to students' homes, and their learning experiences were suddenly contextualized in their residential situation and immediate physical learning environment. The present study consequently examines the role of physical learning environments on different factors influencing students’ learning when pursuing their study from at home. It contrasts the situation of traditional students in a higher education institution and non-traditional students in an academic continuing education institution, which address target groups with different living conditions and needs in learning support. Data were collected via an online survey sent to students enrolled in these two institutions, with a total of 353 students participating during a timeframe impacted by COVID-related lockdowns. We found that stress and well-being is strongly linked to the quality of the surrounding environment of the learning place, whereas perceived motivation is more strongly related to the quality of the learning place itself. How strongly students are affected by these factors is moderated by their overall socio-spatial context. Academic continuing education students are more resilient to sub-optimal physical learning environment than traditional students. Altering the design of the immediate learning environment consequently can help to mitigate factors that negatively impact students’ well-being and learning motivation, which is particularly important for traditional students, who primarily dedicate their time to pursuing their studies.

## Introduction

The COVID-19 pandemic has had significant impact on universities' teaching operations since the beginning of 2020. One common strategy to mitigate the risk of spreading the virus was to significantly reduce or avoid presence-based teaching formats and substitute them with online-based formats. Students were largely not allowed to physically come to campus and had to participate in their courses from their private learning environments usually located at their primary place of living (Ortiz, 2020; Peimani & Kamalipour, 2021).

In on-campus courses, the primary locus of learning is a shared physical space at the university, which provides equal opportunities for participation to students independently of their residential situation. In contrast, courses that are primarily conducted online move the main locus of learning to a physical setting which is different for each participant (Baticulon et al., 2021; Zhao et al., 2021).

How learning can be affected by physical space has been examined in educational sciences as well as in design and architecture (e.g. Barrett et al., 2013; Baticulon et al., 2021; Chhetri, 2020; Choi et al., 2014; Han et al., 2019; Higgins et al., 2005; Sivunen et al., 2014; Wang et al., 2021; Xiong et al., 2018). Summarizing these studies, the quality of physical space has been shown to have impact on the perceived satisfaction, achievement and engagement of learners and in general is considered to be “crucial to learning” (Alphonse et al., 2019). Inadequate workspace and inappropriate equipment can have negative impact on learning (Aguilar & Torres, 2021; Alphonse et al., 2019). Consequently, different physical settings potentially differentiate students' opportunities to achieve satisfying learning outcomes and lead to differences in students’ learning experience, i.e., “anything that promotes learning, including what is observed, felt, heard and done” (Simonson et al., 2019, p. 51), which we here operationalize as the perceived impact of the physical learning environment on motivation to learn, concentration and perceived learning performance.

Research on physical learning environments so far has heavily focussed on their design and effects in institutional settings (i.e., for on-campus learning or in hybrid learning settings, e.g., in (Bülow, 2022). Data on the role of private physical learning environments are limited to date (Alphonse et al., 2019; Zhao et al., 2021). While research on the design of physical learning environments has been pursued already for decades (e.g., Fleming & Storr, 1999; McLaughlin & Mills, 2009), studies on this specific setting are only starting to emerge in the last 2 years, where the main locus of learning processes has shifted from campus to private households during the Corona pandemic (e.g., Cranfield et al., 2021; Ng, 2021; Sonnenschein et al., 2021).

Initial findings on the quality of home learning spaces during the pandemic indicate that “although the learning environment may be virtual, physical space remained vital” (Baticulon et al., 2021, p. 620) Zhao et al. (2021) found that a “comfortable environment for students in terms of temperature, humidity, space light, desk and chair, etc. will help to improve their learning satisfaction” (Zhao et al., 2021, p. 93) in home learning settings. Technical difficulties and distractions, in turn, represent challenges which students face when learning takes place at home (Chhetri, 2020). Distractions appear to mainly arise from other household members or occur due to family responsibilities (ibid.). These household conditions can hinder participation in online courses (Alphonse et al., 2019; Baticulon et al., 2021; Chhetri, 2020; Henaku, 2020; Lassoued et al., 2020; Rotas & Cahapay, 2020). Noise is perceived as particularly disturbing not only in online settings but also during offline learning activities and can affect the concentration during learning (Bringula et al., 2021; Dube, 2020). Dube (2020) found that female students in particular face challenges when engaging in academic work because of household responsibilities. The impact of gender could also be confirmed in our own previous study (Keser Aschenberger et al., 2022), where we examined the perceptions of home learning environments of non-traditional adult students in post-graduate study programs. Data in our study indicate that motivation, well-being, and stress are mainly impacted by the perception of to which extent the learning space (e.g., in terms of technical equipment quality, furniture, and availability of learning space) meets the individual needs (Keser Aschenberger et al., 2022).

It is important to underline that most of the studies mentioned above have been conducted in traditional higher education institutions where participants are mainly full-time students with different needs and living conditions in comparison to continuing education students. However, studies clearly indicate that there exist differences between traditional and non-traditional students on factors related to well-being and learning as stress (Dill and Henley, 1998; Stagman, 2011) and motivation (Johnson et al., 2016). To comprehensively understand the impact factors on students’ learning quality in home learning environments, research thus can benefit from examining diverse sets of students with fundamentally different socio-economic situations and study conditions. In the present study, we therefore set out to examine the impact of physical learning environments on different factors influencing learning of diverse student populations in the context of their studies. Specifically, we address two target groups with distinct living conditions and needs in learning support, students in a traditional higher education institution and an academic continuing education institution (Hannay & Newvine, 2006). Contrasting two types of higher education institutions (HEI) allows to explicitly focus on the differences between students in different stages in life and with diverse socio-economic contexts with respect to the perception and impact of their physical home learning environment.

Consequently, the question guiding our research is

**RQ:** What are the key factors that influence students’ perceptions of their learning processes in a home learning environment?

Operationalizing this question, we examine the differences between students in a traditional higher education institution and an academic continuing education institution regarding their home learning environments during COVID-19 restrictions in terms of

- their physical-spatial conditions including technical equipment,
- their perception of their physical home learning environment,
- the perceived impact of the physical learning environment on the learning experience,
- the impact of “learning place quality” and “indoor environmental quality” and other socio-spatial aspects (such as gender, household structure, availability of learning place) on students’ wellbeing, stress and motivation.

We hypothesize that in particular those aspects, in which the reported perceptions of the examined student populations differ, will be useful to identify, describe and assess the relevant factors of home learning environment quality. A detailed account on these factors allows to examine the differences between physical learning space quality in institutional and private settings. From a practical perspective, it can be useful for universities to address the needs of students with appropriate methodological or organizational measures in settings, where the main locus of learning is at home.

This article is structured as follows: in the next section, we give a brief account on our research design and the methods deployed in our study. We then present the results of the study structured along the analytical dimensions outlined above, and subsequently discuss the results in the light of our research question and prior existing studies. We close with an account on the limitations of our study and further research opportunities.

## Research design and methods

We adopted a cross-sectional survey design for this study (Creswell & Creswell (2018)). Research was conducted in 2020–2021 during the Covid-19 pandemic and the data was collected through an online survey.

### Research context

The study was conducted at two Austrian universities with different educational foci. Johannes Kepler University Linz (JKU) is a regional Austrian university and has four different faculties (engineering and natural sciences; business and social studies; law; and medicine) with around 70 academic degree programs on bachelor-, master- and PhD-level. About 21.000 students are enrolled in these programs, which are mostly offered in traditional full-time formats. Students mostly start their studies there directly after finishing high school and programs are not specifically tailored to allow for traditional employment while studying. Nevertheless, around two thirds of the enrolled students report some sort of (minor) employment aside their studies (Johannes Kepler University, 2019).

The University for Continuing Education Krems (UWK) is a leading European university which is specialised in academic continuing education. In contrast to JKU the UWK focusses solely on post-graduate education, which is organized in three faculties (medicine and health; economics and globalization; and education, arts and architecture) and manifests in over 200 highly specialized master programs. Consequently, students’ socio-demographic structure is different compared to traditional higher education institutions. Students’ average age is around 40, and 19.4% of students are over 50, and 2% are over 60 years. Moreover, a large percentage of the UWK students are employed during their studies and they have several years of work experience including management and leadership. Currently, around 8000 students are registered in the UWK’s programmes.

### Data collection

The questionnaire for the survey was developed through the cooperation of scientists from different disciplines (e.g., education, psychology, and architecture) to investigate the physical home learning environments of students in the academic continuing education sector during the COVID-19 pandemic (initially reported on in Keser Aschenberger et al., 2022).

The questionnaire was originally developed in German, and it consists of four main parts: (1) socio-demographic information and individual physical-spatial conditions of the home learning environment; (2) perceived fulfilment of the personal requirements for the physical learning environment; (3) psychological characteristics like well-being, stress and motivation, measured with standardised questionnaires; and (4) learning experiences during the initial COVID-19-related restrictions. All the variables included in the questionnaire were distilled from the literature, for detailed information on the questionnaire see Keser Aschenberger et al. (2022).

At UWK, the data collection started at the end of June 2020, just after the first wave of COVID-related restrictions to university operations. There was no sampling as all students who were enrolled in courses in the summer semester of 2020 received the link to survey (7737 students). The participation period ended on July 31st. (Results of this round of data collection is presented in Keser Aschenberger et al., 2022).

At JKU, the survey was conducted between the 1st and 22nd December 2020 and thus fell in the timeframe of the second period of COVID-related restrictions. Distribution over university-wide channels was not possible here due to communication policy constraints. To gain a broad sample, students of all faculty were contacted through different online channels organized or supported by the student unions (Discord, Facebook, WhatsApp Groups). These channels are mainly used for helping each other, official announcements, events, and surveys. Hence, they offered a suitable way for distributing the survey to the target group.

### Data analysis

Descriptive statistics were used to gain insight into the UWK and JKU students’ spatial environmental conditions where learning took place during the COVID-19 restriction. Chi^2^ tests were used to investigate the differences between the UWK and JKU students in terms of demographic characteristics, the spatial environmental conditions under which learning took place and the available furniture and technology. An exploratory factor analysis (EFA) was conducted to identify possible dimensions that could explain the interrelationship between the 11 attributes and students’ perception of fulfilled personal requirements of the physical home learning environment. We used independent t-test to analyse the differences between UWK and JKU students regarding the factors of the EFA (F1: Learning Place Quality, F2: Indoor Environmental Quality). The differences between the two groups regarding specific items of F1 and F2 were analysed with Mann–Whitney U tests. We examined the differences between the students regarding the perceived influence of the physical learning environment on their learning experience as well as how they differ on well-being, stress and motivation with independent t-tests. In addition, the impact of personal factors, institution, and physical spatial conditions to predict student’s motivation, stress and well-being was measured using multiple linear regression.

## Results

### Sample characteristics

The online questionnaire was completed by a total of 257 students at the University for Continuing Education Krems (UWK) and by a total of 96 students at Johannes Kepler University Linz (JKU). Table 1 indicates the sample characteristics of the participating UWK and JKU students. In the following, we refer to the participants in our study as “JKU students” or “UWK students” for the sake of clear distinction. Neither term implies that the study allows to derive generalizable statements for the whole population of either university—the present study focusses on comparing the differences between the student groups, which pursue their studies under different framework conditions, rather than comparing the two involved universities in particular.

**Table 1:** Sample demographics and household characteristics of UWK and JKU students

	UWK		JKU		*X*^2^
	*f*	%	*f*	%	
*Gender*^a^ (df = 1, n = 349)					
Male	107	41.6	57	59.4	8.153**
Female	146	56.8	39	40.6	
Diverse	1	0.4	–	–	
No indication made	3	1.2	–	–	
*Age*^a^ (df = 3, n = 351)					
< 21 years	–	–	6	6.3	226.019**
21–25 years	9	3.5	69	71.9	
26–30 years	31	12.1	13	13.5	
> 30 years	215	83.7	8	8.3	
No indication made	2	0.8	–	–	
*Experience with online learning*^a^ (df = 1, n = 353)					
No experience before	140	54.5	51	53.1	.051
Experience before	117	45.5	45	46.9	
*Household form*^a^ (df = 3, n = 349)					
Multi-person household	199	77.4	87	90.6	32.660**
One-person household	50	19.5	–	–	
Shared apartment	4	1.6	7	7.3	
Other household form^c^	–	–	2	2.1	
No indication made	4	1.6	–	–	
*Household structure*^b^ (df = 1, n = 347)					
No children	136	52.9	57	59.4	1.316
Children of compulsory school age	54	21.0	13	13.5	2.484
Children of preschool age	38	14.8	8	8.3	2.525
Children no longer of compulsory school age	23	8.9	7	7.3	.235
Household with more than two generations	16	6.2	12	12.5	3.834
Household with pets	77	30.0	24	25.0	.798
No indication made	5	1.9	3	3.1	.449
*Living environment*^a^ (df = 3, n = 351)					
Urban	85	33.1	41	42.7	3.248
Suburban	63	24.5	23	24.0	
Village environment	72	28.0	23	24.0	
Rural	35	13.6	9	9.4	
No indication made	2	0.8	–	–	
*Residential building type*^a^ (df = 3, n = 335)					
Detached single-family house	108	42	36	37.5	21.230**
Semi-detached or terraced house	19	7.4	7	7.3	
Multi-party house	114	44.4	43	44.8	
Other residential building type^d^	–	–	8	8.3	
No indication made	16	6.2	2	2.1	
*Flat size*^a^ (df = 3, n = 353)					
< 40 m^2^	15	5.8	31	32.3	45.486**
40–70 m^2^	53	20.6	17	17.7	
70–120 m^2^	91	35.4	29	30.2	
> 120 m^2^	98	38.1	19	19.8	
*Access to outdoor spaces*^b^ (df = 1, n = 353)					
Garden	139	54.1	42	43.8	2.988
Terrace	92	35.8	30	31.3	.639
Loggia	106	41.2	38	39.6	.080
No access to outdoor space	37	14.4	27	28.1	8.874**

N = 257 (UWK); N = 96 (JKU)

**p ≤ .01; *p ≤ .05

^a^Only one option to choose, ^b^more than one option to choose, ^c^office building, study room, ^d^student home, office building, two-family house, farm, flat, castle, museum, barrack

Majority of UWK students are female (56.8%), the majority of JKU students are male (59.4%). A Chi^2^-Test showed a significant difference in the genders’ distribution between UWK and JKU, *X*^2^ (1, 349) = 8.153, *p* = 0.004. Further significant differences between UWK and JKU were found for the following characteristics: age, *X*^2^ (3, 351) = 226.019, *p* < 0.001; household form, *X*^*2*^ (1, 349) = 32.660, *p* < 0.001; residential building type, *X*^2^ (1, 335) = 21.230, *p* < 0.001; flat size, *X*^2^ (1, 353) = 45.486, *p* < 0.001, no access to outdoor space *X*^2^ (1, 353) = 8.874, *p* = 0.003.

### Characteristics of home learning environments

In this section of the paper, we present the physical-spatial conditions in which the digitally supported learning of students of UWK and students of JKU took place during COVID-19 restrictions and how these conditions differ.

#### Differences in physical–spatial conditions

The results of chi-square (*X*^2^) tests indicated statistically significant differences between students from UWK and JKU (Table 2) regarding the following items:

**Table 2:** Physical–spatial conditions of home learning environments by university

	UWK		JKU		*X*^2^
	*f*	%	*f*	%	
*Previous existence of learning place*^b^ (df = 1, n = 353)					
Own learning place already available	160	62.3	72	75.0	5.038*
Own learning place newly established	38	14.8	8	8.3	2.568
No specially designated learning place available	72	28.0	16	16.7	4.810*
Learning place also used for other purposes	16	6.2	9	9.4	1.053
*Purpose of the room used for learning*^a^ (df = 1, n = 353)					
Dedicated room for studying	106	41.2	26	27.1	5.988*
Room also used for other purposes	151	58.8	70	72.9	
*Location for learning activities*^a^ (df =1, n = 353)					
Predominantly at designated learning place	179	69.6	71	74.0	.628
Often at other places	78	30.4	25	26.0	
*Availability of learning place*^a^ (df = 1, n = 353)					
Learning place available at all times	192	74.7	75	78.1	.443
Coordination of learning place with others	65	25.3	21	21.9	

N = 257 (UWK); N = 96 (JKU)

**p ≤ .01; *p ≤ .05

^a^Only one option may be chosen, ^b^more than one may be chosen

*Predominantly used learning and workplace* We asked the students at which learning or workplace their learning activities had predominantly taken place since the beginning of the restrictions. We found that more JKU students had a learning place that was already available prior to the pandemic: *X*^2^ (1, 353) = 5.038, *p* = 0.025 (UWK: 62.3%, JKU: 75.0%). Moreover, more UWK students had to study on places that are not designated for learning such as kitchen tables or dining areas compared to JKU students*: X*^2^ (1, 353) = 4.810, *p* = 0.028 (UWK: 28.0%, JKU: 16.7%).

*Purpose of the room used for learning* Concerning the purpose of the room used for studying, we found that 41.2% of UWK students but only 21.1% of JKU students had their own room for studying. For 58.8% of UWK students, the room was also used for other purposes, for JKU students this was the case for 72.9%. The Chi-square test showed a statistically significant difference in the purpose of the room used for learning between UWK and JKU students: *X*^2^ (1, 353) = 5.988, *p* = 0.014.

#### Differences in furniture and IT-equipment

**Fig. 1:**
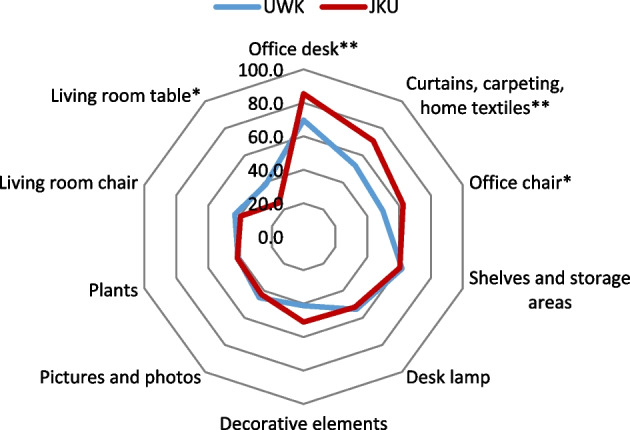
Differences in furniture at home learning places by university

**Fig. 2:**
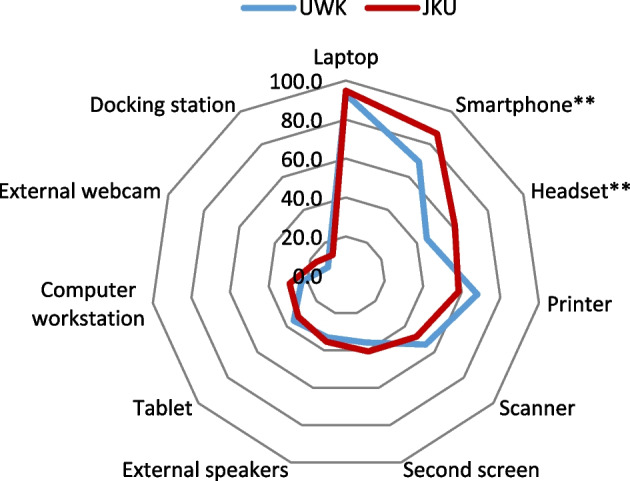
Differences in IT-equipment at home learning places by university

Differences in how the students’ learning places were equipped and what kind of furniture they had were other issues investigated (see Fig. 1). As Fig. 2 shows, IT-equipment was predominantly available for students of UWK and students of JKU. The results of chi-square tests indicated statistically significant differences between the two groups regarding the use of the following items of furniture: office desk, *X*^2^ (1, 353) = 9.017, *p* = 0.003 (UWK: 69.6%, JKU: 85.4%); dining/kitchen table, *X*^2^ (1, 353) = 5.628, *p* = 0.018 (UWK: 38.5%, JKU: 25.0%); office chair, *X*^2^ (1, 353) = 4.525, *p* = 0.033 (UWK: 49.8%, JKU: 62.5%) and curtain/carpets, *X*^2^ (1, 353) = 9.583, *p* = 0.002 (UWK: 52.5%, JKU: 70.8%).

Regarding IT equipment, statistically significant differences were found between UWK and JKU students in the use of smartphones *X*^2^ (1, 353) = 10.727, *p* = 0.001 (UWK: 69.3%, JKU: 86.5%) and in the use of headsets *X*^2^ (1, 353) = 7.097, *p* = 0.008 (UWK: 45.5%, JKU: 61.5%). While 86.5% of JKU students stated to use smartphones at their learning place only 69.3% of the UWK students stated the use of smartphones.

### Perception of home learning environments for digitally supported learning

We examined how students from the two institutions (UWK and JKU) perceived the adequacy of their predominantly used learning environment’s physical and spatial characteristics. We therefore asked students to which extent they agreed that their personal requirements were met at their learning place regarding 11 different attributes on spatial characteristics and indoor environmental conditions. An exploratory factor analysis (EFA) with extraction method of principal component analysis and varimax rotation with Kaiser normalization was used to discover underlying factors. The Kaiser–Meyer–Olkin (KMO) measure of sampling adequacy was 0.843, and Bartlett’s Test of Sphericity was statistically significant (χ2(55) = 1343.55, *p* < 0.001). The EFA produced a two-factor solution (eigenvalues λ > 1), explaining 52.90% of the variance. The results of the factor analysis with factor loadings are presented in Table 3.

**Table 3:** Exploratory factor analysis (EFA) on physical–spatial attributes of home learning environments

	Factor		Degree of Communality
	1	2	*h*_*i*_^*2*^
08 distraction-free environment	**0.78**	0.14	0.63
02 ergonomic work-compatible furniture	**0.77**	0.02	0.59
09 protection against noise pollution	**0.70**	0.30	0.57
11 adaptability to individual spatial requirements	**0.63**	0.39	0.55
01 adequate size	**0.60**	0.41	0.52
03 appropriate technical equipment	**0.56**	0.21	0.36
07 good ventilation conditions	0.19	**0.76**	0.61
04 adequate supply of daylight	0.10	**0.69**	0.49
06 comfortable temperature conditions	0.29	**0.68**	0.55
05 pleasant view	0.13	**0.64**	0.43
10 attractive interior design	0.35	**0.63**	0.52
Eigenvalues λ	3.02	2.80	5.82
Variance explained (%)	27.5	25.4	52.9

The two factors were interpreted as follows:

*Factor 1* Learning place quality (explained 27.5% of variance).

*Factor 2* Indoor environmental quality (IEQ) (explained 25.4% of variance).

The variance in students’ perceptions of the physical learning environment is mainly explained by the "Learning Place Quality" and the "Indoor Environmental Quality".

Internal consistency for each of the scales was examined using Cronbach’s alpha. The alphas were acceptable: 0.81 for Learning Place Quality (6 items) and 0.73 Indoor Environmental Quality (5 items). Table 4 presents the results of the reliability analysis. Furthermore, for the items of the factors, the item selectivity (*rit*) and mean values (*M*) as well as standard deviations (*SD*) were calculated.

**Table 4:** Reliability analysis on F1 and F2

Factors	Items (*k*)	*N*	Cronbach (α)	Selectivity (*r*_it_)	*M* (*SD*)
F1 Learning Place Quality	6	340	0.81	0.46 – 0.64	3.13 (0.87)
F2 Indoor Environmental Quality	5	351	0.73	0.48 – 0.57	3.48 (0.54)

1 = disagree, 2 = rather disagree, 3 = rather agree, 4 = agree (that personal requirements were met);

*N* = listwise deletion

Independent t-tests (Table 5) indicated a statistically significant difference between students of UWK and students of JKU regarding F2 ‘Indoor Environmental Quality’.

**Table 5:** Perceptions on learning place quality and indoor environment quality by university

	UWK		JKU		*t*(df), *p*	Cohen’s *d*
	*n*	*M(SD)*	*n*	*M(SD)*		
F1 Learning Place Quality	256	3.15 (0.68)	95	3.08 (0.61)	0.93 (349), 0.354	–
F2 Indoor Environmental Quality	257	3.53 (0.51)	96	3.36 (0.48)	2.84 (351), 0.005**	0.34

**p ≤ .01

Differences between students of UWK and students of JKU regarding the attributes of F1 ‘Learning Place Quality’ and F2 ‘Indoor Environmental Quality’ were analysed with Mann–Whitney U tests. UWK students had statistically significantly better perceptions of their home learning environment in terms of size, protection against noise pollution, good ventilation conditions, attractive interior design, and pleasant view (cf. Table 6), while JKU students reported statistically significantly better perceptions on the availability of ergonomic work-compatible furniture.

**Table 6:** Different aspects of learning environment by university

	UWK			JKU			*U*	*z*	*p*	Cohen’s *d*
	*n*	*M(SD)*	*Md*	*n*	*M(SD)*	*Md*				
	*F1 learning place quality*									
03 appropriate technical equipment	256	3.52 (0.72)	4.00	96	3.52 (0.67)	4.00	12,113.00	− 0.240	0.810	–
01 adequate size	256	3.42 (0.86)	4.00	96	3.22 (0.89)	3.00	10,542.50	− 2.325	0.020*	0.22
09 protection against noise pollution	255	3.15 (0.94)	3.00	95	2.82 (0.99)	3.00	9828.00	− 2.875	0.004**	0.29
11 adaptability to individual spatial requirements	252	3.15 (0.94)	3.00	92	3.01 (0.94)	3.00	10,572.50	− 1.332	0.183	–
08 distraction-free environment	257	2.99 (1.03)	3.00	96	2.92 (0.93)	3.00	11,545.00	− 0.976	0.329	–
02 ergonomic work-compatible furniture	256	2.70 (1.08)	3.00	96	2.97 (0.95)	3.00	10,680.00	− 1.967	0.049*	0.20
	*F2 Indoor Environmental Quality*									
07 good ventilation conditions	256	3.71 (0.54)	4.00	96	3.54 (0.65)	4.00	10,732.00	− 2.311	0.021*	0.20
04 adequate supply of daylight	257	3.67 (0.62)	4.00	96	3.59 (0.63)	4.00	11,437.00	− 1.1334	0.182	–
06 comfortable temperature conditions	257	3.63 (0.58)	4.00	96	3.52 (0.58)	4.00	10,944.00	− 1.954	0.051	–
10 attractive interior design	257	3.39 (0.78)	4.00	96	3.22 (0.76)	3.00	10,671.00	− 2.147	0.032*	0.21
05 pleasant view	257	3.26 (0.99)	4.00	95	2.92 (1.03)	3.00	9741.00	− 3.161	0.002**	0.32

1 = disagree, 2 = rather disagree, 3 = rather agree, 4 = agree (that personal requirements were met)

**p ≤ .01; *p ≤ .05

### Perceived influence of physical learning environment on learning experience during COVID-19

We further examined how students of UWK and students of JKU perceived the impact of the physical learning environment on their motivation, concentration and learning performance and whether the two student groups differ from their experiences, using independent-samples t-tests. We had five items based on a 7-point rating scale to examines these aspects. Table 7 shows the descriptive data (mean values *M* and standard deviations *SD*) for these items as well as the differences between the two groups. Analyses showed that UWK students statistically significantly differ from JKU students concerning the perceived influence of physical learning environment on motivation (*t*(351) = 2.95, *p* = 0.003, *d* = 0.35), concentration (*t*(351) = 2.33, *p* = 0.020, *d* = 0.28) and learning performance (*t*(351) = 2.70, *p* = 0.007, *d* = 0.32).

**Table 7:** Perceived impact of learning environment on learning experience

	UWK		JKU		*t* (df)*, p*	Cohen’s *d*
	*n*	*M (SD)*	*n*	*M (SD)*		
*Perceived influence of physical learning environment on learning experience*						
Motivation to learn (negative–positive)	257	0.85 (1.71)	96	0.25 (1.69)	2.95 (351), 0.003**	0.35
Concentration (negative–positive)	257	0.65 (1.76)	96	0.17 (1.70)	2.33 (351), 0.020*	0.28
Learning performance (negative–positive)	257	0.81 (1.61)	96	0.29 (1.64)	2.70 (351), 0.007**	0.32

Negative − 3, − 2, − 1, 0, 1, 2, 3 positive

**p ≤ .01; *p ≤ .05

### Influence of physical home learning environment on motivation, stress and well-being

We used the WHO-5 World Health Organisation-Five Well-Being Index for measuring the overall well-being. The average well-being score measured was 14.29 (*SD* = 5.58) for UWK and JKU students, where 0 is the worst possible well-being and 25 is the best possible well-being (13 and below is considered poor well-being). The average stress level measured by Perceived Stress Questionnaire (PSQ) was reported to be 40.44 (*SD* = 21.85), with a range from 0 to 100, and a higher score refers to a higher stress level. Students seem to be less worried compared to the general stress level (*M* = 34.06, *SD* = 25.90), the level of tension was 40.33 (*SD* = 25.85) and the level of demand 48.24 (*SD* = 24.96). Our analysis showed that despite the lockdown, happiness was in the upper average range with 60.95 (*SD* = 24.06). Motivation to learn during the COVID-19 restrictions, measured by the achievement motivation test LEIMO marker items, was reported still high (*M* = 3.90, *SD* = 0.78). Table 8 shows the mean values (*M*) and standard deviations (*SD*) for the questionnaires.

**Table 8:** Means and standard deviations for stress, well-being and motivation (UWK and JKU students)

	*N*	*M (SD)*
WHO-5 well-being	350	14.29 (5.58)
PSQ overall	352	40.44 (21.85)
Worry	349	34.06 (25.90)
Tension	349	40.33 (25.85)
Joy	350	60.95 (24.06)
Demand	349	48.24 (24.96)
LEIMO motivation	344	3.90 (0.78)

Rating scales for the instruments: WHO-5: (0) at no time, (1) some of the time, (2) less than half the time, (3) more than half the time, (4) most of the time, (5) all of the time; PSQ: (1) almost never, (2) sometimes, (3) often, (4) usually; LEIMO: (1) strongly disagree, (2) rather disagree, (3) partly agree (4) rather agree, (5) strongly agree (calculation of mean scores based on the 80% rule)

Furthermore, we also investigated to what extent the students of UWK and the students of JKU differ in their well-being, stress perception and their motivation to learn. The difference was tested using independent-samples t-tests. Statistically significant differences were found in all scales, except for demand. When looking at the mean values, it is noticeable that UWK students have a higher sense of well-being, are more motivated to learn and have a lower stress level compared to JKU students in our sample (Table 9).

**Table 9:** Stress, well-being, and motivation scores by university

	UWK		JKU		*t* (df)*, p*	Cohen’s *d*
	*n*	*M (SD)*	*n*	*M (SD)*		
WHO-5 well-being	254	14.99 (5.46)	96	12.44 (5.51)	3.88 (169.7), < .001**	0.47
PSQ overall	256	37.23 (21.56)	96	48.87 (20.42)	− 4.67 (179.4), < .001**	− 0.55
Worry	254	28.44 (22.37)	95	49.09 (28.70)	− 6.33 (138.9), < .001**	− 0 .85
Tension	254	37.78 (25.68)	95	57.16 (25.19)	− 3.08 (171.6), .002**	− 0.37
Joy	255	64.10 (24.14)	95	52.49 (21.80)	4.30 (185.2), < .001**	0.49
Demand	254	46.94 (25.46)	95	51.72 (23.33)	− 1.66 (182.9), .099	− .19
LEIMO motivation	249	3.99 (.76)	95	3.69 (0.82)	3.10 (159.3), .002**	0.39

Rating scales for the instruments: WHO-5: (0) at no time, (1) some of the time, (2) less than half the time, (3) more than half the time, (4) most of the time, (5) all of the time; PSQ: (1) almost never, (2) sometimes, (3) often, (4) usually; LEIMO: (1) strongly disagree, (2) rather disagree, (3) partly agree (4) rather agree, (5) strongly agree (calculation of mean scores based on the 80% rule)

**p ≤ .01; *p ≤ .05

In order to determine how the physical-spatial conditions influence the motivation, stress and well-being of UWK and JKU students, three hierarchical multiple regression analyses were carried out, with considering gender and institution in a first step. Mean scores of the standardised questionnaires (motivation: LEIMO; stress: PSQ; well-being: WHO-5) were calculated and used as the criteria variables. Predictors were determined as gender (male vs. female); institution (UWK vs. JKU); availability of the learning place (available all time vs. place had to be shared with others); Learning Place Quality (calculated mean score) and Indoor Environmental Quality (calculated mean score). For determining the generalisability of the regression model, we proved if the underlying assumptions have been met. The results show that none of the correlations between predictor variables exceeded the critical value of 0.80 for multicollinearity. The normal distributions of residuals were confirmed (normal curve of histogram; normal probability represents an approximately 45-degree line). The residual terms were independent since the Durbin-Watson coefficient (*d*) was around 2.0 (motivation: *d* = 1.881, stress: *d* = 1.954, well-being: *d* = 1.885).

We entered the predictors into hierarchical regression in two steps. In the first step, gender and institution were entered into regression; in the second step, availability of the learning place, Learning Place Quality and Indoor Environmental Quality were entered into the regression analyses. Table 10 shows the results for each model for each variable.

**Table 10:** Results of regression models of predictors of motivation, stress and well-being

Predictor	Motivation		
*N* = 339	*B*	*SE B*	Β
Step 1 *(R* = .223, *R*^2^ = 5.0%, *R*^2^_adj_ = 4.4%)			
Constant	4.31	0.13	
Gender (female; male)	− 0.21	0.08	− .014*
Institution (UWK; JKU)	− 0.27	0.09	− 0.16**
Step 2 *(R* = .324*, R*^2^ = 10.5%, *R*^2^_adj_ = 9.1%)			
Constant	3.12	0.40	
Gender (female; male)	− 0.21	0.08	− 0.14*
Institution (UWK; JKU)	− 0.24	0.09	− 0.14*
Availability of learning place	0.04	0.11	0.02
F1 Learning Place Quality (score)	0.22	0.08	0.19**
F2 Indoor Environmental Quality (score)	0.13	0.10	0.08

	Stress		
*N* = 347	*B*	*SE B*	β
Step 1 (*R* = .240, *R*^2^ = 5.7%, *R*^2^_adj_ = 5.2%)			
Constant	39.41	3.54	
Gender (female; male)	− 1.55	2.29	− 0.04
Institution (UWK; JKU)	11.90	2.60	0.24**
Step 2 (*R* = .400, *R*^2^ = 16.0%, *R*^2^_adj_ = 14.7%)			
Constant	72.53	10.73	
Gender (female; male)	− 1.84	2.24	− 0.04
Institution (UWK; JKU)	10.55	2.49	0.22**
Availability of learning place	5.39	2.83	0.11
F1 Learning Place Quality (score)	− 4.19	2.20	− 0.13
F2 Indoor Environmental Quality (score)	− 7.45	2.70	− 0.17**

	Well-being		
*N* = 345	*B*	*SE B*	β
Step 1 (*R* = .201, *R*^2^ = 4.0%, *R*^2^_adj_ = 3.5%)			
Constant	14.93	0.92	
Gender (female; male)	0.03	0.59	0.003
^Institution^ (UWK; JKU)	− 2.52	0.67	− 0.20**
Step 2 (*R* = .384, *R*^2^ = 14.8%, *R*^2^_adj_ = 13.5%)			
Constant	3.56	2.77	
Gender (female; male)	0.15	0.58	0.01
Institution (UWK; JKU)	− 2.08	0.64	− 0.17**
Availability of learning place	− 0.50	0.73	− 0.04
F1 Learning Place Quality (score)	1.29	0.57	0.15*
F2 Indoor Environmental Quality (score)	2.20	0.70	0.20**

Criteria = motivation, stress, well-being; Predictor Step 1 = institution, gender; Predictor Step 2 = availability of learning place at all times; factor 1 ‘Learning Place Quality’; factor 2 ‘Indoor Environment Quality’

Coding: Gender: 1 = female, 2 = male, 3 = diverse; Institution: 0 = UWK; 1 = JKU; Availability of learning place: 1 = available all time; 2 = had to be shared with others

**p < .01; *p < .05

Regression analysis for motivation shows a multiple correlation coefficient of *R* = 0.223 between the linear combination of the two predictors gender and institution. Model 1 statistically significantly predicts the motivation to learn, *F*(2,336) = 8.77, *p* =  < 0.001, *R*^2^ = 0.050, *R*^2^_adj_ = 0.044. The combination of these two predictors accounts of 4.4% of the variation in motivation to learn. According to standardized coefficients (β), there is a negative correlation between gender and motivation as well as between institution and motivation. This result indicates that the motivation to learn is higher for female students and UWK students. In Model 2, the multiple correlation coefficient between the linear combination of three predictors, the combination of availability of learning place, Learning Place Quality and Indoor Environmental Quality and motivation, increased to *R* = 0.324 after controlling for the effects of gender and institution. Model 2 statistically significantly predicted motivation, *F*(5,333) = 7.79, *p* =  < 0.001, *R*^2^ = 0.105, *R*^2^_adj_ = 0.091. The combined factors of availability of learning place, Learning Place Quality and Indoor Environmental Quality accounted for 9.1% of the variance in motivation above gender and institution. According to standardised coefficients (β), there is a still a negative relationship between gender, institution, and motivation to learn. Furthermore, Learning Place Quality showed a positive impact on motivation to learn (β = 0.19).

For stress, the multiple correlation coefficient between the linear combination of the two predictors gender and institution is *R* = 0.240. Model 1 statistically significantly predicted stress perception, *F*(2,344) = 10.49, *p* =  < 0.001, *R*^2^ = 0.057, *R*^2^_adj_ = 0.052. The combination of these two predictors accounts for 5.2% of the variation in stress perception. According to standardised coefficients (β), there is a positive relationship between the institution and stress (β = 0.24), which may indicate that stress perception is higher for JKU students in our sample. In Model 2, the multiple correlation coefficient between the linear combination of three predictors, i.e., the combination of the availability of the learning place, Learning Place Quality and Indoor Environmental Quality, and stress increased to *R* = 0.400 after controlling for the effects of the gender and institution. Model 2 statistically significantly predicted stress, *F*(5,341) = 12.96, *p* < 0.001, *R*^2^ = 0.160, *R*^2^_adj_ = 0.147. The combined factors of availability of learning place, Learning Place Quality and Indoor Environmental Quality accounted for 14.7% of the variance in stress controlling gender and institution. According to standardised coefficients (β), there is a still a positive relationship between institution and stress (β = 0.22); and a negative relationship between Indoor Environmental Quality and stress (β = − 0.17).

Regarding the well-being, analysis shows a multiple correlation coefficient of *R* = 0.201 between the linear combination of gender and institution, and well-being. Model 1 statistically significantly predicted well-being, *F*(2,342) = 7.20, *p* = 0.001, *R*^2^ = 0.040, *R*^2^_adj_ = 0.035. The combination of these two predictors accounts for 3.5% of the variation in well-being. According to standardised coefficients (β), there is a negative relationship between the institution and well-being (β = − 0.20), which indicates a lower well-being for JKU students. In Model 2, the multiple correlation coefficient between the linear combination of three predictors, i.e., the combination of the availability of the learning place, Learning Place Quality and Indoor Environmental Quality, and stress increased to *R* = 0.384 after controlling for the effects of the gender and institution. Model 2 statistically significantly predicts stress, *F*(5,339) = 11.75, *p* < 0.001, *R*^2^ = 0.148, *R*^2^_adj_ = 0.135. The combined factors of availability of learning place, Learning Place Quality and Indoor Environmental Quality accounted for 13.5% of the variance in well-being above gender and institution. Standardised coefficients (β) show a negative relationship between institution and stress (β = − 0.17); and a positive relationship between Learning Place Quality (β = 0.15) and Indoor Environmental Quality and well-being (β = 0.20).

In this research, it was explored how well spatial characteristics such as the availability of the learning place, Learning Place Quality and Indoor Environmental Quality predict motivation, stress, and well-being. We also analysed the role of gender and institution. Results showed that spatial characteristics explained 9.1% in motivation, 14.7% in stress, and 13.5% in well-being after controlling for gender and institution.

In all models the institution shows an impact on motivation to learn, stress perception and well-being. Learning Place Quality shows a similar impact on motivation (β = 0.19) and on well-being (β = 0.15). The same applies to the influence of Indoor Environmental Quality, here it also shows a highly similar impact on stress (β = − 0.17) and on well-being (β = 0.20).

## Discussion

The aim of the present study was to examine the impact of physical learning environments on different factors influencing learning of diverse student populations. The study was conducted at two HEIs with fundamentally different target groups to diversify the student populations the sample was drawn from. The analysis of the socio-demographic data collected in the study confirms that the living conditions of students from these two institutions differ fundamentally (**RQa**): Students of the traditional university (JKU) live in significantly smaller flats and have significantly more limited access to open space than students at the continuing education university (UWK).

Furthermore, the residential building type of the living environment differs significantly between UWK and JKU students, with UWK students living significantly more often in detached single-family-houses and JKU students also stating that they live in other residential building types, such as student homes. The availability of an explicitly designated learning space, however, was significantly higher for students at JKU, which is also reflected in the available equipment, where higher number of JKU students had access to office desks and chairs. Such equipment, however, was often located in a room that was also used for other purposes than studying, which is in line with the more spatially constrained living conditions. Overall, the data confirms the different physical-spatial study conditions these two group of students encounter during learning from home.

While JKU students have access to objectively better equipped workplaces than UWK students in terms of both furniture, and technology relevant for study activities, the perceived overall learning place quality (**RQb**) does not differ significantly among the students of the two institutions (except for the aspect of noise-protection at the learning place, which show significantly worse results for JKU students). The perceived indoor environment quality was rated significantly higher by UWK students than by JKU students, which is in line with the objectively more comfortable living conditions of UWK students (more spacious, more often in single-family-houses – the item showing the largest perceived advantage for UWK students is “pleasant view”).

UWK students’ more positive perceptions of their physical-spatial conditions also seems to translate to higher well-being and higher positive impact of the learning environment on the overall learning experience (**RQc**). UWK students reported that their learning environment during the restriction had a significantly more positive impact on their motivation, concentration, and overall perceived learning performance than it did for JKU students. While not entirely surprising, this is a noteworthy finding that confirms the importance of good environmental conditions and learning place infrastructure on students’ learning experience as also pointed out in prior research (e.g. Alphonse et al., 2019). Some notable differences in the data of the two samples point at the aspects that seem to be particularly relevant: the available physical space, ergonomic furniture and in particular protection against noise seem to distinguish the perceived quality of the immediate learning place for the two examined groups. Noise is also identified as a particularly relevant impact factor in prior studies (Bringula et al., 2021; Dube, 2020). Good ventilation, attractive interior design and in particular a pleasant view make a difference in terms of perceived indoor environmental quality.

When looking in more detail at which factors explain differences in students’ motivation, perceived stress, and well-being in their home learning environment during COVID-19-related home study activities (**RQd**), our study showed that, overall, stress and well-being are significantly positively impacted by a high perceived indoor environmental quality (i.e., the aspects characterizing the surrounding environment). Similarly large effects could be found for the institution the students belonged to—UWK students felt less stressed and reported higher well-being than JKU students. Based on the different socio-economic characteristics of the examined student populations, we can hypothesize that—besides the fact that UWK students in general also report higher perceived indoor environmental quality—might have benefited more strongly from a switch to distance learning, as they could reduce or avoid time-consuming traveling to campus and thus be able to better balance their studies with their other obligations (such as work and family), which might have led to reduced perceived stress and improved well-being—a phenomenon for which evidence also has been found in prior studies (Berry & Hughes, 2020; Hannay & Newvine, 2006; Kibelloh & Bao, 2014). Traditional students might have less experience with needing to balance different resource-demanding situations and thus might have been more severely impacted by the fundamental change of study conditions caused by COVID-19 (Chung et al., 2017). Other impact factors, in particular gender, do not have any significant impact on perceived stress and well-being. As for gender, this somewhat contrasts the findings of Dube (2020). They, however, attribute disadvantages to higher amounts household chores, which might have not been prevalent in the sample examined in the present study.

Motivation, in contrast to stress and well-being, has a higher correlation to learning place quality (i.e., the quality of the immediate learning place and its equipment). Indoor environmental quality (i.e., the quality of the space surrounding and contextualizing the immediate learning place) does not significantly contribute to variations in motivation. The important role of learning place quality is in line with earlier diagnoses from literature, where workspace and equipment quality were found to affect learning (Aguilar & Torres, 2021; Alphonse et al., 2019; Aristovnik et al., 2020; Raaper et al., 2021). Summarizing the role of the physical–spatial learning environment, motivation to learn in a home learning setting is highly correlated to the availability of a high-quality workplace, where a distraction-free environment, ergonomic furniture and noise protection are the most relevant impact factors, and less impacted by the quality of the environment surrounding the learning place.

Overall, the study could confirm and deepen the understanding of role of the physical-spatial learning environment for well-being and students’ perceived learning experience in home-learning settings. The specific focus of the study to explore the differences between distinctive HEI sectors (traditional universities vs. continuing education) and their specific student populations has allowed to identify the role of different impact factors on the perception of home learning environment quality. Even though the quality of immediate workplace is not perceived to differ significantly between the examined populations, motivation to learn, well-being and stress differ significantly based on the institutional affiliation. Continuing education students usually need to balance the resource demands of their studies with more other obligations (such as work and family) that traditional students (Lowe & Gayle, 2007; Maharani et al., 2020; Rockinson-Szapkiw & Watson, 2020). They thus might have benefited more strongly from a switch to distance learning, where increased flexibility and less travel times might have contributed more strongly to reduce perceived stress as well as improve well-being and motivation to learn than for traditional students. These advantages appear to even compensate more challenging settings regarding the availability of a dedicated learning place and its equipment. Students at a traditional university, in contrast, appear to be more sensitive to challenges of the home learning situation itself (higher stress, lower subjective well-being) and report less influence of the physical–spatial setting in which they study.

## Conclusions

In this article, we have examined the role of physical learning environments on different factors influencing students’ learning in their home learning processes. We explicitly focused on student populations with different socio-economic backgrounds and study conditions and could confirm the difference between the impact of the immediate learning place quality and the quality of the surrounding environment, which we have already identified in a previous study (Keser Aschenberger et al., 2022). The data set obtained from a diverse student population allowed to examine these and additional potential other influencing factors in more detail. The present results show that perceived stress and well-being is strongly linked to the quality of the surrounding environment of the learning place, whereas perceived motivation is more strongly related to the immediate learning place quality. In comparing the different student populations, we could further differentiate the impact of variations in these factors. How strongly students are affected by factors of the physical environment which negatively impact their learning experience (such as lack of a dedicated learning place or sub-optimal equipment) is moderated by their overall socio-spatial context. Continuing education students, who are usually used to having to balance the resource demands of different obligations in life, appear to be more resilient to sub-optimal learning place experience than traditional students. The perceived deficiencies had a stronger effect on traditional students’ learning experience, even if their immediate workplace quality is objectively higher than that of the former group. Summarizing, we can conclude that the physical-spatial properties of the home learning environment have significant impact on perceived stress, well-being and motivation, but the strengths of effects vary in relation to the overall socio-economic living conditions of a learner. Consequently, interventions in the design of the immediate learning environment can help to mitigate factors that negatively impact learning motivation, which is particularly important for students, whose main focus in life is pursuing their studies. The environment surrounding the learning place has a strong impact on perceived stress and well-being. These findings provide relevant information for the future development and design of private but also institutionally provided learning spaces for different student groups considering their differing context situations and requirements. Our findings can support individual learners to create an optimised home learning environment, which can help to reduce the sense of stress and increase well-being. However, as our study shows, the individual framework conditions differ a lot and possibilities for optimisation are likely to be limited. So from an institutional perspective, it is important to recognise the differences in students’ living conditions and needs, and consequently provide access to on- or off-campus learning spaces that mitigate the effects of inequality identified in this study. Furthermore, our findings can provide important foundations for the future design of learning spaces in student residences and publicly accessible facilities, such as libraries or co-learning spaces. Finally, our findings can contribute to inform the change process in the housing sector induced by the digital transformation through an increased need for suitable learning and workplaces in the home environment.

As with the majority of studies, the design of the current study is subject to limitations. The results are based on participants' statements regarding their learning environment and how home learning environments influenced motivation, stress and well-being during the first COVID-19 constraints. Since a convenience sampling approach including two institutions was followed for the survey and with a smaller return rate, the results have limited statistical representativeness for the overall population and potentially also have to be interpreted in the light of a self-reporting bias and the time that has passed since data collection.

In the context of future research, the investigation of private learning spaces of students at universities could be extended geographically and also to learners in the non-academic education sector. Furthermore, the suitability of publicly accessible off-campus learning spaces (e.g. public buildings, squares, parks, transportation, …) could be analysed. In addition to surveys, other research methods such as image documentation, diary entries, etc. could be used.

## Data Availability

Not applicable.

## Abbreviations

EFA: Exploratory factor analysis
F1: Factor 1
F2: Factor 2
JKU: Johannes Kepler University Linz
KMO: Kaiser–Meyer–Olkin
LEIMO: Achievement motivation test (German: Leistungsmotivationstest)
PSQ: Perceived Stress Questionnaire
UWK: University for Continuing Education Krems
WHO-5: World Health Organisation-Five Well-Being Index

## References

[CR1] Aguilar, M. V., & Torres, G. (2021). Making sense of online classes during quarantine due to the COVID-19 pandemic: Students’ perceptions from a Philippine University. *Asia Social Issue,**14*, 248066–15.

[CR2] Alphonse, A., Orellana, A., & Kanzki-Veloso, E. (2019). How online students describe their physical learning environment. *Quarterly Review of Distance Education,**20*(2), 27.

[CR3] Aristovnik, A., Keržič, D., Ravšelj, D., Tomaževič, N., & Umek, L. (2020). Impacts of the COVID-19 pandemic on life of higher education students: A global perspective. *Sustainability,**12*(20), 8438. 10.3390/su12208438

[CR4] Barrett, P., Zhang, Y., Moffat, J., & Kobbacy, K. (2013). A holistic, multi-level analysis identifying the impact of classroom design on pupils’ learning. *Building and Environment,**59*, 678–689. 10.1016/j.buildenv.2012.09.016

[CR5] Baticulon, R., Sy, J., Alberto, N., Baron, M., Mabulay, R., Rizada, L., Tiu, C., Clarion, C., & Reyes, J. (2021). Barriers to online learning in the time of covid-19: A national survey of medical students in the Philippines. *Medical Science Educator,**31*, 1–12. 10.1007/s40670-021-01231-z PMC7904236 33649712

[CR6] Berry, G. R., & Hughes, H. (2020). Integrating work–life balance with 24/7 information and communication technologies: The experience of adult students with online learning. *American Journal of Distance Education,**34*(2), 91–105.

[CR7] Bringula, R., Reguyal, J. J., Tan, D. D., & Ulfa, S. (2021). Mathematics self-concept and challenges of learners in an online learning environment during COVID-19 pandemic. *Smart Learning Environments,**8*(1), 1–23. 10.1186/s40561-021-00168-5 PMC8520328 40477819

[CR8] Bülow, M. W. (2022). Designing synchronous hybrid learning spaces: Challenges and opportunities. In E. Gil, Y. Mor, Y. Dimitriadis, & C. Köppe (Eds.), *Hybrid learning spaces. Understanding teaching–learning practice. *Cham: Springer. 10.1007/978-3-030-88520-5_9

[CR9] Chhetri, C. (2020). ‘ I Lost Track of Things’ student experiences of remote learning in the Covid-19 pandemic. In *Proceedings of the 21st annual conference on information technology education* (pp. 314–319).

[CR10] Choi, H.-H., van Merriënboer, J. J. G., & Paas, F. (2014). Effects of the physical environment on cognitive load and learning: Towards a new model of cognitive load. *Educational Psychology Review,**26*(2), 225–244. 10.1007/s10648-014-9262-6

[CR11] Chung, E., Turnbull, D., & Chur-Hansen, A. (2017). Differences in resilience between ‘traditional’ and ‘non-traditional’ university students. *Active Learning in Higher Education,**18*(1), 77–87. 10.1177/1469787417693493

[CR12] Cranfield, D. J., Tick, A., Venter, I. M., Blignaut, R. J., & Renaud, K. (2021). Higher education students’ perceptions of online learning during COVID-19—A comparative study. *Education Sciences,**11*(8), 403.

[CR13] Creswell, J. W., & Creswell, J. D. (2018). *Research design: Qualitative, quantitative, and mixed methods approaches* (5th ed.). SAGE.

[CR14] Dill, P. L., & Henley, T. B. (1998). Stressors of College: A comparison of traditional and nontraditional students. *The Journal of Psychology,**132*(1), 25–32. 10.1080/00223989809599261 9447723 10.1080/00223989809599261

[CR15] Dube, M. C. (2020). Online learning challenges postgraduate certificate in education history students faced during COVID-19 at the University of Zululand. *Yesterday and Today,**24*, 136–157.

[CR16] Fleming, D., & Storr, J. (1999). The impact of lecture theatre design on learning experience. *Facilities,**17*(7/8), 231–236. 10.1108/02632779910270186

[CR17] Han, H., Moon, H., & Lee, H. (2019). Physical classroom environment affects students’ satisfaction: Attitude and quality as mediators. *Social Behavior and Personality: An International Journal,**47*(5), 1–10. 10.2224/sbp.7961

[CR18] Hannay, M., & Newvine, T. (2006). Perceptions of distance learning: A comparison of online and traditional learning. *Journal of Online Learning and Teaching,**2*(1), 1–11.

[CR19] Henaku, E. A. (2020). COVID-19 online learning experience of college students: The case of Ghana. *International Journal of Multidisciplinary Sciences and Advanced Technology,**1*(2), 54–62.

[CR20] Higgins, S., Hall, E., Wall, K., Woolner, P., & McCaughey, C. (2005). *The impact of school environments: A literature review*. Design Council.

[CR21] Johannes Kepler University. (2019). Gender & Diversity Bericht 2019. Johannes Kepler University. https://www.jku.at/abteilung-personalentwicklung-gender-diversity-management/referate/referat-gender-diversity-management/.

[CR22] Johnson, M. L., Taasoobshirazi, G., Clark, L., Howell, L., & Breen, M. (2016). Motivations of traditional and nontraditional college students: From self-determination and attributions, to expectancy and values. *The Journal of Continuing Higher Education,**64*(1), 3–15. 10.1080/07377363.2016.1132880

[CR23] Keser Aschenberger, F., Radinger, G., Brachtl, S., Ipser, C., & Oppl, S. (2022). Physical home learning environments for digitally-supported learning in academic continuing education during COVID-19 pandemic. *Learning Environments Research*. 10.1007/s10984-022-09406-0 10.1007/s10984-022-09406-0 PMC8867450 35228831

[CR24] Kibelloh, M., & Bao, Y. (2014). Can online MBA programmes allow professional working mothers to balance work, family, and career progression? A case study in China. *The Asia-Pacific Education Researcher,**23*(2), 249–259.

[CR25] Lassoued, Z., Alhendawi, M., & Bashitialshaaer, R. (2020). An exploratory study of the obstacles for achieving quality in distance learning during the COVID-19 pandemic. *Education Sciences,**10*(9), 232.

[CR26] Lowe, J., & Gayle, V. (2007). Exploring the work/life/study balance: The experience of higher education students in a Scottish further education college. *Journal of Further and Higher Education,**31*(3), 225–238.

[CR27] Maharani, A., Intan, S., Mahlani, S. A., & Berlian, C. W. (2020). Flexible working arrangement, stress, worklife balance and motivation: Evidence from postgraduate students as worker. *Jurnal Organisasi Dan Manajemen,**16*(2), 196–213.

[CR28] McLaughlin, P., & Mills, A. (2009). Where shall the future student learn? Student expectations of university facilities for teaching and learning. *Synergy,**7*(1), 8–13.

[CR29] Ng, C. F. (2021). The physical learning environment of online distance learners in higher education—A conceptual model. *Frontiers in Psychology,**4130*, 635117. 10.3389/fpsyg.2021.635117 PMC8506005 34650464

[CR30] Ortiz, P. A. (2020). Teaching in the time of COVID-19. *Biochemistry and Molecular Biology Education,**48*, 201. 32239800 10.1002/bmb.21348 PMC7228309

[CR31] Peimani, N., & Kamalipour, H. (2021). Online education and the covid-19 outbreak: A case study of online teaching during lockdown. *Education Sciences,**11*(2), 72.

[CR32] Raaper, R., Brown, C., & Llewellyn, A. (2021). Student support as social network: Exploring non-traditional student experiences of academic and wellbeing support during the Covid-19 pandemic. *Educational Review*. 10.1080/00131911.2021.1965960

[CR33] Rockinson-Szapkiw, A., & Watson, J. H. (2020). Academic-family integration: How do men and women in distance education and residential doctoral programs integrate their degree and family? *Online Learning,**24*(4), 112–130.

[CR34] Rotas, E. E., & Cahapay, M. B. (2020). Difficulties in remote learning: Voices of Philippine University students in the wake of COVID-19 crisis. *Asian Journal of Distance Education,**15*(2), 147–158.

[CR35] Simonson, M., Zvacek, S. M., & Smaldino, S. (2019). *Teaching and learning at a distance: Foundations of distance education* (7th ed.). Information Age Publishing.

[CR36] Sivunen, M., Viljanen, J., Nenonen, S., & Kajander, J.-K. (2014). Evidence-based design in learning environments: A practical framework for project briefing. *International Journal of Facilities Management,**13*, 162–174.

[CR37] Sonnenschein, S., Stites, M., & Ross, A. (2021). Home learning environments for young children in the US during COVID-19. *Early Education and Development,**32*(6), 794–811.

[CR38] Stagman, D. (2011). A comparison of traditional and nontraditional college students' stress and its relationship to their time management and overall psychological adjustment. HIM 1990–2015. 1185. https://stars.library.ucf.edu/honorstheses1990-2015/1185.

[CR39] Wang, C., Zhang, F., Wang, J., Doyle, J. K., Hancock, P. A., Mak, C. M., & Liu, S. (2021). How indoor environmental quality affects occupants’ cognitive functions: A systematic review. *Building and Environment,**193*, 107647. 10.1016/j.buildenv.2021.107647

[CR40] Xiong, L., Huang, X., Li, J., Mao, P., Wang, X., Wang, R., & Tang, M. (2018). Impact of indoor physical environment on learning efficiency in different types of tasks: A 3 × 4 × 3 full factorial design analysis. *International Journal of Environmental Research and Public Health,**15*(6), 1256. 10.3390/ijerph15061256 29899260 10.3390/ijerph15061256 PMC6025257

[CR41] Zhao, L., Hwang, W.-Y., & Shih, T. K. (2021). Investigation of the physical learning environment of distance learning under COVID-19 and its influence on students’ health and learning satisfaction. *International Journal of Distance Education Technologies,**19*(2), 77–98. 10.4018/IJDET.20210401.oa4

